# A Rare Case of Metastatic Hepatocellular Carcinoma Masquerading as a Forehead Hematoma

**DOI:** 10.1155/2020/8842936

**Published:** 2020-09-30

**Authors:** Kimberly Sanders, Ashley Thomas, Carmen Isache, Anwer Siddiqi

**Affiliations:** ^1^Department of Internal Medicine, University of Florida College of Medicine Jacksonville, Jacksonville, FL, USA; ^2^Department of Pathology, University of Florida College of Medicine Jacksonville, Jacksonville, FL, USA

## Abstract

Hepatocellular carcinoma (HCC) is the most common primary liver cancer and can arise from any form of chronic liver disease or cirrhosis. With increasing rates of metabolic syndrome and obesity, it is not surprising that NASH is quickly becoming a leading cause of chronic liver disease and HCC in the western hemisphere (Wang and Malhi, 2018). Metastasis is usually found in advanced stages of the disease, owing to its poor prognosis. The lung, bone, and lymph nodes are the most frequent sites of metastasis (Balogh et al., 2016, and Becker et al., 2014). On the other hand, metastasis to the skin and cranium is relatively rare. Literature review reveals less than 10 reported cases in the last 10 years. Herein, we report an unusual case of a “forehead hematoma” leading to the formal diagnosis of metastatic HCC.

## 1. Introduction

Hepatocellular carcinoma (HCC) is a leading cause of morbidity and mortality with increasing incidence worldwide [1]. What was formerly thought of as a malignancy predominantly affecting Eastern Asians [2] has broken racial barriers and border lines to affect almost all ethnicities and countries alike. Men in their fourth to fifth decades are more commonly affected than women. Its incidence has been increasing in the US and Western Hemisphere, quickly becoming one of the highest causes of death and ultimately increasing the economic healthcare burden [2, 3]. Therefore, it is imperative for primary care physicians, gastroenterologists, and internists to screen for HCC in at-risk populations. The majority of HCC cases are due to cirrhosis or chronic liver disease from alcohol abuse or viral infection from hepatitis B or hepatitis C [3]. Hepatitis B accounts for 44% of HCC cases, the majority of which arise in Asia, while hepatitis C accounts for 21% of cases worldwide [4]. As expected with increasing rates of diabetes and obesity, nonalcoholic steatohepatitis (NASH) is starting to account for a large percentage of HCC cases in the US, with an annual incidence of 2.6% and rising [5]. Diabetes and metabolic syndrome alone increase the risk for HCC by up to 2 fold [5], reflecting the importance of patient counseling and screening.

Early stages of HCC are largely asymptomatic, leading to a delay in presentation. As a result, HCC is usually diagnosed at advanced stages, owing to its poor prognosis and median survival <20 months [5]. The presence of metastasis at the time of diagnosis is even more dismal. Approximately 5–15% of patients have evidence of metastasis at the time of diagnosis and 30–50% of patients develop metastasis at some point in the disease process, owing to the aggressive nature of this cancer [6]. The lung is the most common site of metastasis, followed by lymph nodes and bone [7]. Bony metastases have a predilection to the spine, pelvis, and ribs [8]. However, metastases to the skull, as well as skin and soft tissue, are relatively rare.

## 2. Case Presentation

**Figure 1:**
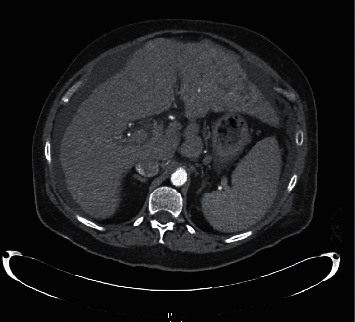
CT liver mass protocol with IV contrast showing evidence of cirrhosis with features of portal hypertension, an ill-defined infiltrative mass in left hepatic lobe suggestive of infiltrative HCC, hypoattenuating lesions in the right hepatic lobe, and periportal lymphadenopathy.

**Figure 2:**
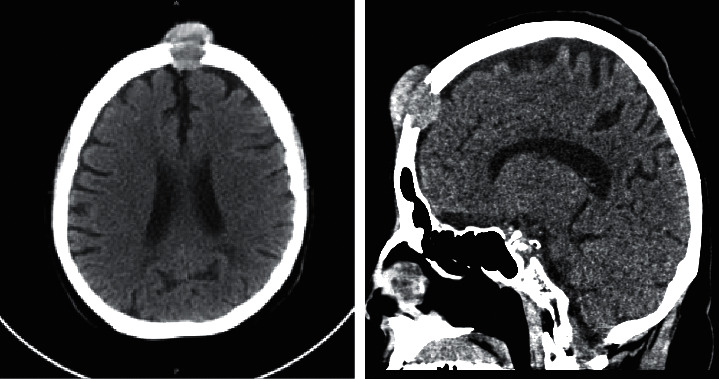
Cross-sectional and sagittal images of CT head (with and without contrast) demonstrating a 3.1 cm × 3 cm × 3 cm enhancing soft tissue mass eroding through the frontal calvarium and extending into the anterior soft tissues of the scalp in the epidural space overlying the left frontal lobe.

A 77-year-old female with a past medical history of hypertension was admitted for new onset ascites and decompensated cirrhosis. She presented to the emergency room for progressively worsening shortness of breath, abdominal distention, and 10-pound weight loss over 4 weeks. Incidentally, the patient was also noted to have a painless soft tissue mass on her forehead. She reported that the lesion had been enlarging over 4 months which she thought to be secondary to a hematoma that developed after hitting her head on a door. She denied prior history of alcohol abuse. There was no personal or family history of liver disease, cancer, or autoimmune diseases. Vital signs were stable, with a BMI of 26. Physical exam was notable for significant abdominal distention, palpable hepatosplenomegaly, and a fixed nontender firm mass on her forehead. No encephalopathy, asterixis, or stigmata of liver disease was appreciated. Cardiopulmonary exam was unremarkable without crackles or peripheral edema. Initial labs demonstrated thrombocytopenia and a mild transaminitis. INR and bilirubin were within normal limits. Computed tomography (CT) of the abdomen and pelvis revealed large-volume ascites, splenomegaly, and cirrhotic liver morphology with multifocal hyperattenuating liver lesions, the largest measuring 6 cm × 5 cm × 5 cm involving segments II-II with extracapsular extension, associated with porta hepatis and both mesenteric and anterior mediastinal lymphadenopathy. Findings were highly suspicious for metastatic hepatocellular carcinoma (Figure 1). CT head revealed a 3.1 cm mass eroding through the frontal calvarium (Figure 2). Further investigations were significant for hemoglobin A1c (7.4%) and hypertriglyceridemia (201 mg/dL). Chronic liver disease workup was otherwise unremarkable including viral hepatitis serologies (HAV, HBcAb, HBcAg, and HCV Ab), ceruloplasmin, ferritin and transferrin, ANA, C-ANCA, P-ANCA, mitochondrial antibody, anti-smooth muscle antibody, and IgG. Serum alpha feto protein (AFP) was elevated at 97.9 ng/ml. Transthoracic echocardiogram was not performed. Diagnostic paracentesis revealed SAAG (serum to ascites albumin gradient) >1.1 and low total protein, consistent with portal hypertension. Fluid cytology was negative for malignant cells.

**Figure 3:**
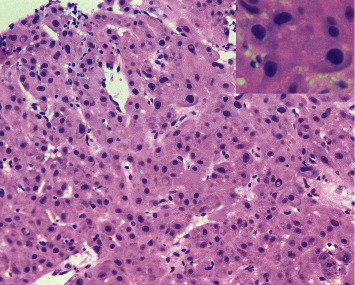
H&E 10*×* section shows well to moderately differentiated HCC with trabecular pattern. The inset (H&E 20*×*) shows large pleomorphic nuclei with nucleoli (histopathology of forehead lesion).

**Figure 4:**
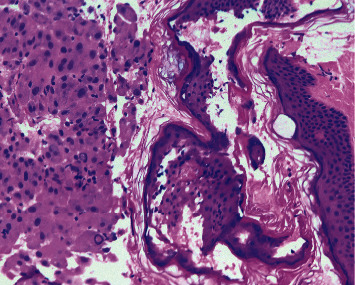
H&E 10*×* showing the forehead skin fragment as a contaminant on the right and the tumor on the left (histopathology of forehead lesion).

**Figure 5:**
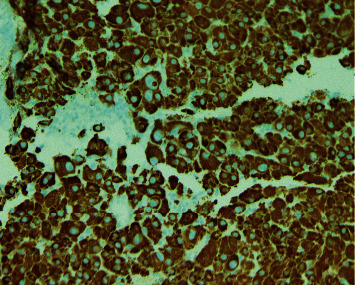
Hep Par-1 stain positive (histopathology and immunostaining of forehead lesion).

**Figure 6:**
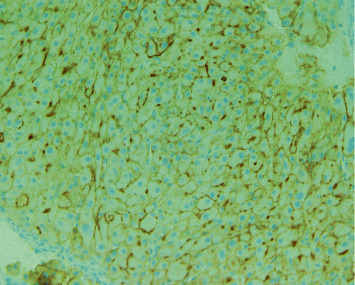
Positive polyclonal CEA staining, diffuse canalicular pattern (histopathology and immunostaining of forehead lesion).

CT liver mass protocol demonstrated ill-defined infiltrative mass in left hepatic lobe (segments 2 and 3 extending to segments 4A and 4B) and multiple hypoattenuating lesions in the right hepatic lobe concerning for multifocal HCC but no significant arterial phase enhancement or delayed venous washout. Due to poorly delineated margins, the masses were unable to be measured accordingly. As imaging was indeterminate and there was a high clinical suspicion for HCC, a biopsy was necessary to confirm the diagnosis. Given the location and accessibility, a core needle biopsy of the forehead lesion was performed to provide pathologic diagnosis and identify the presence of metastasis, which would ultimately determine treatment. Pathology revealed a well to moderately differentiated tumor with trabecular pattern. Morphologically, these tumor cells resemble hepatocytic cells with slight atypia (Figures 3 and 4). No bile production was noted on the limited biopsy. A positive Hep Par-1 staining confirmed the lineage of these cells to be hepatocellular in origin (Figure 5). Polyclonal CEA staining (Figure 6) demonstrated a diffuse canalicular pattern, highly suggestive of HCC, rather than cytoplasmic staining as seen in cholangiocarcinoma and many other cancers. AFP staining was negative. Other types of immunohistochemistry with arginase or keratin 7 or 20 were not performed. At our institution, these stains are reserved for situations in which the metastatic tumor cells are poorly differentiated or if Hep Par-1 is negative.

The patient was classified as Child-Pugh class B, given her large volume ascites, absence of encephalopathy, total bilirubin <2 mg/dL, albumin 3 g/dL, and INR <1.7. However, due to evidence of metastasis on imaging, she was deemed a poor liver transplant candidate-based Milan and USCF criteria. She was managed medically with diuretics for ascites and was discharged home to follow-up with oncology for palliative chemotherapy. Unfortunately, the patient succumbed to her disease before the initiation of any treatment and passed away 2 months later.

## 3. Discussion

Hepatocellular carcinoma (HCC) is one of the few cancers that can be diagnosed without tissue biopsy [1] but by multiphase contrast-enhanced CT or MRI. In cirrhotic or high-risk patients, the diagnostic criteria includes size ≥1 cm, arterial phase enhancement, and a combination of washout, capsule enhancement, or a 50% interval increase in size ≤6 months [9]. If imaging is inconclusive, a tissue biopsy can be pursued. In noncirrhotic patients or in cases in which the primary tumor is unknown, a liver biopsy is necessary to diagnose HCC. Characteristic histological features include wide trabeculae of more than 3 cells, increased nuclear-cytoplasmic ratio, unpaired arteries, and loss of reticulin network [9]. However, histopathology alone is insufficient to establish the diagnosis as a number of tumors can mimic the morphologic features of HCC, especially in metastasis. Immunohistochemistry with Hep Par-1, heat-shock protein 70 (HSP70), arginase-1, and glypican-3, among many others, aids in the differentiation of these tumors and improves the diagnostic yield. For example, glypican-3, HSP70, and glutamine synthetase staining can distinguish HCC from high-grade dysplastic nodules in the liver [10]. However, these particular staining methods were not performed in our patient as the biopsy was obtained from an extrahepatic site and, therefore, the differentiation of HCC from a high-grade dysplastic nodule was not warranted. This patient's biopsy revealed well-differentiated tumor cells resembling hepatocytes with atypia, and its lineage was confirmed with positive Hep Par-1 staining. Furthermore, polyclonal CEA staining of this cranial lesion showed a diffuse canalicular pattern, highly suggestive of HCC. In contrast, cholangiocarcinoma and many other cancers demonstrate a cytoplasmic pattern on polyclonal CEA staining. At our institution, arginase and keratin 7 and 20 are reserved for poorly differentiated metastatic tumor cells or negative Hep Par-1 staining.

Early screening and recognition for HCC allows for multiple treatment options and therefore improved survival outcomes. Management involves a multidisciplinary approach, and selection is based on tumor size, location, liver function, and presence of extrahepatic spread. Orthotopic liver transplantation (OLT) is the definitive treatment of HCC. Unfortunately, as earlier stages of HCC are largely asymptomatic, most patients present with advanced disease and, therefore, are not candidates for OLT based on the Milan criteria (up to three liver lesions ≤3 cm or a single lesion ≤5 cm, in the absence of macrovascular invasion or extrahepatic metastasis) [11]. Metastatic disease, although limited in its therapeutic options, can be treated with sorafenib, an oral tyrosine kinase inhibitor. Sorafenib works on a variety of different receptors and mechanisms to inhibit tumor angiogenesis and proliferation [12]. It is reserved for advanced HCC with preserved liver function in those who are not surgical candidates or have failed other locoregional therapies. For years, sorafenib was the only treatment option for these patients with a median survival of less than three months [13]. Since 2017, advances have been made in targeted immunotherapy with several new drugs demonstrating noninferiority to sorafenib and a median survival of one year [13]. And most recently, immunotherapy with checkpoint inhibitors (CPIs) have demonstrated promising results in treatment responses in patients with advanced HCC, prompting a number of clinical trials to evaluate this potential therapeutic benefit [14].

Cranial and cutaneous metastases of HCC are relatively rare and even more unusual to be the primary presentation of HCC. This patient exhibited evidence of metastasis on her forehead several months before developing any features of cirrhosis. In the absence of significant alcohol consumption and after exclusion of other causes, the etiology of her cirrhosis is presumed to be from NASH in the setting of unrecognized diabetes mellitus and hyperlipidemia. However, without proper histopathological examination of the liver, NASH is indistinguishable from cryptogenic cirrhosis (which includes the progression of NASH to “burnt out NASH”). This case illustrates the rare locations HCC can metastasize to but, also, reflects the importance of a thorough history and physical examination, keeping a vigilant eye for abnormal lesions that should be investigated to rule out malignancy. Early recognition of this patient's enlarging forehead mass could have lead an earlier diagnosis, which may not have changed her overall poor prognosis but may have allowed for improved quality of life by preventing decompensation with early palliation.

## 4. Conclusion

Hepatocellular carcinoma is an aggressive tumor that most commonly arises in the background of chronic liver disease and cirrhosis from alcohol abuse, viral hepatitis, NASH, or several other conditions that affect the liver. It can be largely preventable with community education on minimizing risk factors, proper screening, vaccination against hepatitis B, and early treatment of at-risk populations, i.e., screening for and treating hepatitis C or NASH, for example. Routine screening for HCC in liver cirrhosis patients has made it possible to identify and treat the tumor in its early stages. However, many are diagnosed at advanced stages, with low survival rates at two years of diagnosis. Extrahepatic metastases are rarely the primary presentation of HCC, but it is important to hold high clinical suspicion of an underlying primary malignancy, as early diagnosis may lead to improved quality of life with early treatment or palliation.

## Data Availability

The data used to support the findings of this study are included within the article.
